# German Teachers’ Digital Habitus and Their Pandemic Pedagogy

**DOI:** 10.1007/s42438-020-00174-9

**Published:** 2020-08-12

**Authors:** Carolyn Blume

**Affiliations:** Institute of English Studies, Leuphana Universität Lüneburg, Universitätsallee 1, 21335 Lüneburg, Germany

**Keywords:** Covid-19, Digitalization, Teacher professionalization, Habitus, Germany

## Abstract

After closing public schools in early 2020 to slow the spread of Covid-19, attempts to provide continuity of education in Germany by means of digital tools faltered in variety of ways, with insufficient competence and inadequate technology leading to inequitable access and uneven implementation. Understanding how German teachers were caught unprepared in this time of crisis, especially in comparison with their European neighbors, requires an examination of their habitus as discussed by Bourdieu and Wacquant (1992) that accounts for their behaviors beyond existing models regarding technology acceptance. Drawing on existing sociological and media-related studies, this contribution will describe the attitudes of German teachers and educational decision-makers in light of their digital, cultural, and educational habitus to provide a partial explanatory account for the current state of affairs. It will show how traditional skepticism for innovation among teachers in general, and German teachers in particular, is reinforced by demographic and sociological characteristics of the German teacher population and the nature of German schooling. After describing extant conditions regarding digitally mediated educational experiences during the initial Covid-19 phase in Germany based on emerging data, this article will subsequently identify prospective issues in this area in the near future. While the transition to digital teaching and learning has the potential to bring about a number of challenges, early data suggests that a possibility of significant positive development may occur as well. Based on these indications, the article will conclude with implications for teacher professionalization going forward.

## Introduction

While the emergence of Covid-19 disrupted all facets of daily life around the world, education was one of the sectors most severely affected by the sudden imperative to move teaching and learning from primarily face-to-face interactions to distanced structures. Within weeks, and with little public discussion, digitally supported teaching and learning at primary, secondary, and tertiary levels was established in many countries. Beyond this initial impulse, however, there have been few pedagogical, philosophical, or organizational consistencies in how this shift has transpired, both internationally and at a national or even local level. In Germany, even the label for this type of learning is inconsistent, with many continuing to call the practices that have taken hold ‘digital learning,’ despite the fact that many observers argue that ‘(emergency) remote learning’ or ‘blended learning’ are more accurate descriptors (Kerres 2020). Still others distinguish the latter of these from the ‘hybrid learning’ that has accompanied the partial re-opening of schools.

Regardless of the terminology, most analyses indicate that these practices have been implemented in ways that are pedagogically and structurally problematic, revealing the degree to which German teachers and learners—as well as decision-makers at institutional and policy levels—were ill-equipped to master this shift, with issues of multiple hindrances to access compounding inadequate competence among both teachers and students (Arp et al. 2020; Eickelmann and Drossel 2020). Numerous examples of poorly photographed worksheets, students unable to manage learning platforms, and uncurated lists of websites flooded social media in the first phase, giving way after the initial shock to queries regarding video conferencing and the creation and dissemination of explainer videos and ‘webinars’ in subsequent weeks. Pervading all of this were concerns regarding data privacy and the exclusion of learners with limited digital access. Given the well-documented inadequate degree of digitalization in German schools (Eickelmann et al. 2019), this state of affairs is not surprising. However, the reasons for this situation, and the implications of these factors in terms of forthcoming pedagogy and policy, remain incompletely elaborated.

This article will begin by summarizing some of the existing research that seeks to explain the teachers’ reluctance to adopt digital tools, in light of both empirical and theoretical models, which has given rise to the current situation. It will then focus on an examination of the demographically informed habitus of Germany’s teachers to elucidate their reservations regarding digitally mediated instruction before considering the school habitus itself as a factor in this constellation. This background will shed light on the current status quo in German primary and secondary schools before considering possible scenarios for the future and concluding with an analysis of the implications for teacher training going forward.

## Teachers and Innovation Rejection

A number of theories exist to explain teachers’ adoption or rejection of digital technology, many of which have been applied to consider the special case of German schoolteachers (Blume 2020b; Drossel and Eickelmann 2018; Karimzadeh et al. 2017; Kommer and Biermann 2012; Mayer and Girwidz 2019). Although these analyses rely on decades of international scholarship on the subject, the combination of factors and their relative significance remain necessarily unclear. In a recent blog post, Cuban (2020) argues that the current state of affairs reflects a range of unknown factors that make each teaching situation unique, and cautions against blaming teachers for the diversity of approaches. Drawing a parallel to the radically different outcomes in virus-related fatalities, he asks, ‘Why the differences? Geography? Culture? Demography? Luck? No one in authority or any expert can say with confidence.’ While Cuban posed his questions in relation to what he saw in the USA specifically, he identifies a range of factors that likely account for a number of the digital permutations that are being practiced elsewhere as well. These reflect those factors that mediate teacher adoption of innovation more generally; pedagogical tradition, disciplinary silos, time constraints, limited opportunities to engage in professional communities of practice, and lack of agency and autonomy all influence the incremental pace of change in schools, including the absent digital revolution (Cuban et al. 2001; Ketelaar et al. 2012; Windschitl and Sahl 2002). These are measured in such constructs as technology acceptance and adoption models (Koehler and Mishra 2009; Petko 2012), categorized according to teacher typologies (Drossel and Eickelmann 2019; Ehmke et al. 2004), and analyzed in light of self-efficacy, age, and gender (Drossel et al. 2019).

That teachers in Germany specifically are not favorably inclined to using digital media is well documented. In comparison with practitioners in other countries, German teachers have been shown to integrate digital media in their instruction less frequently than twelve out of thirteen countries that participated in the most recent international study on the topic (Eickelmann et al. 2019). Whereas the international average of teachers engaging in regular classroom usage was determined to be 78.2% of all teachers, the German average in 2018 was 60.2% (Eickelmann et al. 2019). While these percentages do not indicate the type or quality of digital media usage being carried out, the numbers alone indicate that there is a significant difference between German teachers and their international counterparts. Moreover, accumulating evidence suggests that there are also distinct national differences between German students studying to become teachers (henceforth: pre-service teachers) and students in other subject areas. Compared with their peers, pre-service teachers are less likely to utilize digital media, want to integrate e-learning, and identify competencies pertaining to digital media (Kerres 2003; Schmid et al. 2017).

The reasons for all of these disparities are difficult to ascertain, and have been considered from a variety of approaches. While Kerres (2020) emphasizes the structural factors shaping teachers digital (non-)usages in light of the current situation, other studies in the past have focused on teachers’ beliefs. Given the fact that pre-service teachers not yet teaching demonstrate less digital affinity than their similarly situated peers, the institutional factors associated with schools cannot fully explain these differences. Thus, it is clear that something else must play a role in determining this non-usage. Although analyses of teachers’ beliefs have long played a role in explaining their digital behaviors, as demonstrated by thorough qualitative (Ertmer and Ottenbreit-Leftwich 2010; Tour 2015) and quantitative (Mayer and Girwidz 2019; Nistor et al. 2014; Rohs et al. 2020; Rubach and Lazarides 2019) studies, examinations of these factors have not brought about a substantial change in the status quo.

## Habitus of Teachers

Despite the wealth of research into teachers’ beliefs, few studies have focused on the role played by teachers’ personal habitus, i.e., their constitutive dispositions that shape their attitudes towards, and usages of, digital technologies (Bourdieu and Wacquant 1992). This is surprising, given the influence ascribed to habitus in myriad ways, the studies that consider the relevance of learners’ familial milieus to understand school achievement (Lange-Vester and Vester 2018), the literature on the habitus of schools generally (Bremer 2009; Budde 2013; Nohl 2007), and how the habitus of schooling may affect digital usages more specifically (Brüggemann and Welling 2009). Moreover, the few studies that do focus on examining teachers’ beliefs through the lens of their habitus clearly demonstrate the ways in which these shape their attitudes towards (digitally mediated) teaching and learning (Graham 2008; Grubesic 2013; Meurer 2006), thus highlighting the importance of habitus to understanding digitally mediated pedagogy.

Furthermore, although the habitus of German teachers appears, in many ways, to be relatively homogeneous, this is a superficial analysis that requires closer examination in order to understand. Approximately 70% of German teachers come from middle to upper cultural and economic strata (Cramer 2010), reinforcing Bourdieu’s (2001) premise that contemporary school culture is oriented towards the conventions of these middle and upper classes for which and by whom they were constructed (Lange-Vester and Vester 2018). At the same time, a more nuanced examination reveals that within this initially coarse class-based classification system, teachers actually stem from the variety of socioeconomic and sociocultural milieu (ibid.) that are associated with the dissolution of standardized relations between class and norm (Mikos 2007). Thus, the reasons for their relatively consistent attitudes towards cultural attitudes and digital phenomena, which could be construed as communities of taste (Mutsch 2012), demand further examination, especially in light of the pedagogical implications of these attitudes.

Bourdieu’s theory of habitus accounts for the attitude of a particular individual, as a member of a social group, towards an object that will help determine how it will actually be used, regardless of the potential uses said object may possess, facilitate, or enable. These underlying attitudes are shaped by dispositions that stem from deeply held and largely implicit values and are projected onto technologies, which Sterne (2003: 376) describes as ‘little crystallized parts of habitus.’ In his analysis of televised news, for example, Bourdieu (1996) emphasizes how it is the norms of journalism and of the academy, as much as the technology itself, that shapes how information is presented and how it is perceived. He goes on to point out that the distaste of academics for the superficial presentations found on television have less to do with the medium, and more to do with the ‘enabling and constraining conventions of the journalistic field’ (Sterne 2003: 373). While Bourdieu focused on France, it is worth noting that the German disdain of intellectuals towards ‘television’ (cf. Mikos 2007) is partially reproduced in the disdain conveyed by the contemporary teaching class towards digital media that is the focus of this article. Although some users see a difference between the passivity of the television and the interactivity of the computer (Friedrichs et al. 2016), for many, it is now not news that is vilified, but non-serious usages of computers, tablet, mobile devices, and their accout rements (Henrichwark 2009; Meurer 2006; Mutsch 2012). Despite the fact there is some evidence of sophisticated digital media usage among those with ‘elite’ capital, entertainment-heavy usages often lead their users feeling embarrassed by their pursuit of supposedly insignificant activities (Mikos 2007; Mutsch 2012; Niesyto 2009). However, this single disposition alone cannot suffice to explain teachers’ attitudes towards and usages of newer technologies, and it would be remiss to suggest that the medium makes no differences at all (Krommer 2018b). Other ‘tastes,’ in the Bourdiean sense, relating to art, for example, as well as more fundamental notions of creative production (Pileggi and Patton 2003) and uses of leisure time (Robson 2009), equally inform these preferences that Sterne refers to as ‘embodied social knowledge that may or may not be conscious’ (Sterne 2003: 375). Most directly, ‘tastes’ regarding pedagogical norms also influence the instructional use of media (Belland 2009).

In Germany, these attitudes or tastes that can best be described as skeptical of many usages of digital media can be traced back to several sources. Mikos (2007) focuses on the tradition of the Enlightenment, which established information and *Bildung* as the primary sources of valid capital, without explicating these more fully. Stern (1974), however, does just this, and draws a connection to the modern era, arguing that German humanism reflects ‘a moral command… This command, the essence of what the Germans often called individualism, could best be followed by the pursuit of culture, by literary and esthetic education. The idealism of the later nineteenth century embodied an exceptional veneration for learning, for the cultivation of the self’ (xxiv). Stern continues by highlighting the impact of this attitude: ‘At its best, this veneration inspired the dedicated energy of Germany’s scholars; at its worst… [it added] a powerful rationalization into the already formidable barrier between the educated and the uneducated classes’ (xxv). This disparagement of other kinds of types of capital is realized in the highly stratified educational system that continues to exist in Germany today, in which college-preparatory *Gymnasia* enjoy substantial prestige over other school forms (cf. Kaiser 2002) and in which homogeneity of classes is (still) the idealized norm (Reh 2005). Lepenies (2009) provides a further reason for Germany’s infatuation with so-called highbrow culture and delineates continuations from the nineteenth century to the late twentieth: where national unity and political normalcy have been a challenge, elite cultural production has provided an alternative. Widening the gap between the upper classes and the ‘rest,’ Hill (2008) argues, with a glance at the Frankfurt School, is the notion that the agentive nature of cultural offerings is reserved for this type of production. Non-elites are thus not entitled to access to such agency, nor is it supposedly accessible in their own forms of culture.

This chasm between the ‘educated and the uneducated classes’ may help explain orientations towards cultural practice in Germany today, although ‘uneducated’ in a high-wealth country such as Germany (Fantom and Serajuddin 2016) with universal education needs to be replaced with descriptors referring to less-valued socioeconomic and sociocultural capital. As Katz-Gerro (2002) shows, while the middle classes in Israel, Sweden, and the USA orient their tastes, informed by habitus, towards those adopted by the so-called lower classes, the opposite is true in Italy and Germany. Here, the ‘middle classes,’ from which the majority of teachers in Germany are drawn, pattern their preferences on that of the upper classes. Despite increasing plurality of cultural consumption in which class and preferences are complexified, the tastes of Germany’s middle classes continue to be primarily informed, not by popular culture, but by ‘highbrow culture’ (Katz-Gerro 2002). Thus, despite the fact that the teaching profession in Germany is seen as one that is relatively open towards welcoming individuals from lower socioeconomic and sociocultural strata, it is at the same time largely unfamiliar with the habits and culture of those groups. Nor are these ‘foreign cultures’ valued. Yet it is in ‘popular’ (and youth) culture where digital lives thrive: where social media is used as both as tool for networking and performativity (Niesyto 2009); where digital gaming opens doors to skilled communities of practice (Blume 2019); where video platforms challenge the role of newspapers and television broadcasts to inform and influence (Breunig et al. 2014; Davies 2015).

Research into third level digital divides confirms these distinctions: usage patterns of digital technology have been shown to differ according to ethnicity, gender, education, migration experiences, and wealth in a variety of national settings (Borg and Smith 2018; Cruz-Jesus et al. 2016; Livingstone and Helsper 2016; Mertens and d’Haenens 2010), including Germany (Lutz 2016; Richter et al. 2019; Senkbeil et al. 2019; Zillien and Hargittai 2009). Although Pakulski and Waters (1996) argue that consumption styles, rather than occupational classes, define social markers in wealthy societies, it is simultaneously true that occupational classes share similar consumption styles based on habitus-informed tastes. Why this might be more so the case among German teachers than in other countries or professions can only be speculated on, but may have to do with the habitus of culture and schooling, the former of which is discussed previously and the latter of which will follow, which reproduce these preferences. In the terminology of ‘will, skill, tool’ model (Petko 2012), it becomes clear that the habitus-informed ‘will’ of the teaching class to partake in many of these digital practices is absent, with the ‘skills’ they accumulate representing only a small portion of the kinds of media practices that could inform educational practices in heterogeneous settings, and a utilitarian sense of the ‘tools.’ Finally, while the personal habitus does not fully determine professional attitudes, it does influence them (Bolten 2018; Meurer 2006; Mutsch 2012).

The potential usages of the technological medium are mediated by the users’ attitudes on the one hand and the affordances of the object on the other. Mikos (2007), referring again to television, points out that the more generic the offerings of the tool, the more attractive it is to a wider group of users. At the same time, this generic nature both enables and obligates the individual to customize the medium to their tastes. These customizations, which include the ways in which digital media are produced and received, represent the various forms of capital which lend those who possess them stature within specific communities (Mikos 2007; Niesyto 2009). Thus, the use of the Internet to parse current events, play digital games, write blogs, adapt memes, or manage financial affairs is a personalization of the generic medium that both reflects and constitutes capital, establishes social fields, and is the basis for distinctions that construct communities (Henrichwark 2009; Niesyto 2009). It is thus also a critical site for mediating identity and for demonstrating and perpetuating habitus (Davies 2015).

Only a few studies focus specifically on these dispositions, which Kommer and Biermann (2012) refer to as the *mediale habitus*. Their studies focus on the habitus specifically among pre-service teachers. Although this population apparently accepts the use of digital media for informational or professional activities, they perpetuate a comparatively conservative set of beliefs as regards other usages of digital media. More than half of the pre-service teachers the authors surveyed recalled their parents’ condescending attitude towards computers in general, and especially towards computer games, gaming consoles, cellphones, and private television programming. Other studies have found activity patterns among (pre-service) teachers as regards to digital game playing specifically that reflect these attitudes (Blume 2020b; Mutsch 2012). Elsewhere, the primacy of conventional habitus is evident when, for example, teachers express concerns that new media forms could marginalize more traditional ones or even content itself (Bockermann 2014). Meurer (2006) demonstrates how elementary school teachers construct ‘learning’ and ‘computers’ as diametrically opposed to one another, and all forms of computer usage as unproductive activities among less educated families with little redeeming value. Although these findings were not replicated in a small-scale study by Bockermann (2014), she did find a comparatively high degree of anxiety around the idea of using digital media in formal learning environments because of its potentially negative effects on instruction. These dispositions can be traced back to the general habitus of the respondents’ familial milieu, which values classical forms of leisure and entertainment on the one hand and views computers as purely informational tools on the other (Kommer and Biermann 2012). This dichotomy is accompanied by denigration of digital habits that are less ‘refined.’ In this way, this population uses their tastes to underscore the distance between themselves and other milieu (Pakulski and Waters 1996).

## Habitus in Schools

While the habitus of German schools as a place where middle- and upper-class norms are constituted and reproduced is well-documented in terms of both its existence and its negative impact on learners who do not demonstrate the respective capital (Kramer and Helsper 2011; Lange-Vester and Vester 2018; Maaz et al. 2011), these analyses do not necessarily explain the continued lack of meaningful digital usages. As described in the previous section, a closer examination of teachers’ personal habitus provides some context and, as will be shown in this section, can be utilized to illuminate aspects of German school habitus that mitigate against a wider acceptance of digital media. The elements of German school culture discussed below are not comprehensive analyses of German schools, but they do show how teachers’ predominant habitus relegates digital media to the pedagogical sidelines.

Habitus-informed differences notwithstanding, German school teachers represent a relatively homogeneous group in comparison with the students they encounter (Nohl 2007). Identifying with an academic milieu, the teachers construct distinctions between themselves and their learners (Bremer 2009). Corresponding to a ‘black-and-white’ tendency predominant among this milieu (Meurer 2006), this attempt to maintain authority leads to a rejection of many attitudes, objects, and activities that could serve to establish similarities between themselves and their students. Instead, schools perpetuate the distance between teachers and students, which manifest itself in a variety of ways. At an abstract level, this is not difficult to do, as the familial habitus of German teachers described previously differs from that of many students, creating natural lines of difference between the two groups. At a pragmatic level, this is seen in the continued reluctance to accord contemporary literacy forms or interests validity within the classroom or curricula. In its most extreme forms, this contributes to a perspective that deems students’ tastes as deficient (Bremm and Racherbäumer 2020). To do otherwise would be to relinquish both authority over what constitutes valid cultural and symbolic capital, as well as authority over who makes these determinations (Grau 2009; Hill 2008).

An example of this process can be seen in the existential concerns for education voiced by various educational theorists of the last two generations. After delineating the reasons for the widening gap between students’ worlds and school worlds, which can be primarily found in (digital) popular culture, Ziehe (2007) highlights the challenges posed by students’ interaction forms, interests, and ways of knowing. What is lost, he argues, is not just the legitimacy of ‘high’ culture as an umbrella for all of society, but also students’ self-control, shown in an inability to fulfill obligations, concentrate adequately, or manage disinterest. The outcome, he foresees, is an increasing lack of distinction between students’ extramural worlds and educational norms, climaxing in the institution’s ‘cognitive suicide for fear of death due to a lack of motivation’ (Ziehe 2007: 111, translation author). Giesecke (1995), while arguing that schools cannot continue to treat mass media, and leisure and consumption choices as the enemy, likewise argues that only a clear distancing of school culture from these modes of interaction can address the prevalent lack of discipline and seriousness of purpose: ‘The task of schooling cannot be to reproduce the maxims of the “entertainment society” within its walls, television can do that better. Instead, it must emphasize the idea of enlightening instruction in opposition to all other, extramural expectations of the students and no less, of the parents: only then can it work in cooperation with other socializing factors to play its unique role’ (Giesecke 1995: 99, translation author). He goes on to emphasize that, within schools, the norms that exist must be made clear: ‘There are no powerless social institutions, the question is always only *whose* power will be recognized *with what legitimation*’ (101; italics in the original; translation author). Schools must use this authority to convey ‘middle-class language’ as something that is not arbitrary, but as the legitimate form of public communication. While thus claiming to value youth language equally to ‘school language,’ he makes simultaneously it clear that the former is invalid.

The forces that uphold these attitudes and processes are enacted by teachers in a number of ways, who arguably seek to distinguish themselves from ‘the masses’ (Bremer 2009). By focusing on the low degree of ‘affinity-seeking’ behavior of German teachers, for example, Roach and Byrne (2001) make it clear that German teachers actively construct difference between themselves and their pupils. This is further reinforced by the ways in which teachers treat learners’ interests with skepticism. Grau (2009), focusing on English language activities in and out of school, attests to the fact that the two are ‘worlds apart’ (160). Outside of school, the ninth graders in her study engage in English via music, television and cinema, the Internet, and computer games. The teachers’ estimation of the number of incidences their learners engage in with each of these categories deviates significantly from the numbers reported by learners, with teachers generally underestimating the role of traditional media and contact overall with English outside of school. However, the teachers overestimate the students’ use of digital media for communicative and game playing purposes while underestimating the number of English language texts the students read online (165). While the use of English online is itself a topic that bears close analysis beyond the scope of this paper, the teachers’ presumptions seem to reinforce their image of learners as interested only in entertainment. Moreover, they reject the use of learners’ authentic texts as supposedly inappropriate for educational purposes—a position supported by 12% of the learners themselves. Overall, the results reinforce Bockermann’s (2014) findings that many teachers know little about what their students actually do online, and only a minority see these activities as meaningful. This rejection of authenticity aligns with what Katz-Gerro (2004: 14) describes as a process of marking distinction: ‘Analysis of cultural consumption should consider all cultural forms as potential cultural resources exchangeable in different markets. Nevertheless, appreciation of cultural forms is unevenly distributed and may reflect and reinforce domination and exclusion. For example, Meyer (2000) distinguishes the rhetoric of authenticity and the rhetoric of refinement as two types of taste formation. The rhetoric of refinement alludes to symbolic meanings that are the result of an aristocratic configuration of power. The rhetoric of authenticity refers to taste formed in the context of sweeping challenges to aristocratic privilege and symbolic practice.’

The emphasis on ‘industriousness’ (Kaiser 2002) and the rejection of ‘fun’ (Roeder 2001) in German schools invalidates the authentic interests of learners, including those that might, especially in terms of digital literacies, be considered playful (Blume 2020b). Thus, those youth from better situated and better educated families who use digital media in ways that align more closely to that of the teachers, valuing digital tools for their informational and communicative affordances, are more likely to gain their teachers’ recognition than those learners from less advantaged backgrounds whose digital competencies focus on opportunities for ‘entertainment’ (Henrichwark 2009; Niesyto 2009). This occurs, among other reasons, to assert the authority of the middle-class values espoused by teachers who are, in the words of Niesyto (2009), caught up in their own *Bildungsbürgertum*, or bourgeoisie notions of what constitutes knowledge, and fearful of the codes that could replace it. Bockermann (2014) found that over 40% of the pre-service teachers she surveyed anticipated that their students might know more about digital media than their teachers, and that such a discrepancy could threaten their perceived role as teachers. The problem is not merely the digital incompetence of the teachers, but their inability to value their students’ varied and complex, and sometimes sophisticated, digital media competencies that leads to superficial critiques and analyses of the medium (Niesyto 2009). In order to maintain this distinction, a denigration of digital tools and their pedagogical place in the institution of schooling occurs.

## Digital Teaching in a Time of Corona

While the reasons for German (pre-service) teachers’ limited usage of digital media is only partially explained by an analysis of their habitus and that of their schools, this perspective helps shed light on the problems faced by all participants—teachers, learners, and parents—in the wake of the closure of schools in March of 2020. In the following, some of the problems, gleaned from emerging studies and dialogues, will be highlighted in light of their relevance to their aforementioned issues of habitus. Given the emerging nature of the situation, the data do not always rely on the stringent requirements usually applied to scholarly analysis (cf. Fickermann and Edelstein 2020). There is significant concern, especially, that the perspectives of families and students from lower socioeconomic strata are underrepresented in many of the early surveys and data collections that have been, due to the nature of the situation, carried out in an online environment, precisely where these individuals and communities reside less frequently (cf. C. Huber 2020). However, the data relied upon here describe issues that have been identified by individuals and educational researchers and are indicative of trends that necessitate further empirical examination. Finally, with the perspective focused specifically on differences in habitus, issues of inequitable participation are centered, notwithstanding the nature of the available data.

### Economic Capital

While the preceding analysis of teachers’ and learners’ habitus does not examine issues of economic capital, that is, the material possession of access to digital tools, it is clear that these issues are relevant in terms of the current situation. With 90% of teachers relying on private digital tools to establish and maintain contact with their students (GEW 2020a), there is little evidence that material access among individual teachers is inadequate. However, this statistic does suggest that the material availability of digital media in schools is inadequate, leaving educators to invest their own resources in hardware and software. Although the question allows for various interpretations, only one-third of teachers in a representative study indicate that their school was well prepared to use digital media prior to the closing of schools (Eickelmann and Drossel 2020), suggesting that both infrastructure and didactic planning were limited. In terms of learning management systems, only a little more than one-quarter of the teachers were able to access one for their students during the spring of 2020 (Eickelmann and Drossel 2020), significantly fewer than those in Switzerland and Austria (S. G. Huber et al. 2020).^1^ In April 2020, Lower Saxony introduced its own learning management system, which was found in May 2020 to have significant data privacy concerns and was thus temporarily shut down (NDR1 2020). On the face of it, this data reveals little about the digital attitudes of teachers. However, it does indicate—despite a heralded digital investment program implemented in 2019 (GMK 2019)—a prior lack of conceptual commitment towards the digitalization of German schools by school leaders, themselves teachers, and policymakers, who arguably possess an analogous set of beliefs leading to similar attitudes, both of which stem from similar habitus. Simply put, the personal digital attitudes of school teachers and school administrators meant that no one in German schools or who is responsible for their funding or development prioritized digitalization. Without widespread advocacy, digitalization has been neither promoted nor funded.

Equally problematic for the implementation of digitally mediated schooling during this period has been the well-publicized inequity between learners with and without digital resources. Although the studies have documented that the rate of smartphone ownership among German teenagers is over 90%, this number drops to 50% among children between the ages of six and 12, i.e., those in primary and some early secondary grades. Rates of laptop, computer, or tablet ownership are significantly lower (between 25 and 67%) (Feierabend et al. 2018). In addition to questions as to whether a cell phone is an adequate device with which to access educational materials, participate in video conferences, or communicate with peers and teachers, it is likely that, while some children and teens possess multiple devices, others have limited, shared, or no access to digital hardware (Huber et al. 2020; Kramer 2020).

Finally, access to hardware alone is inadequate, as any digital tool is only useful if it allows for interaction via the Internet. While fewer than 9 million out of 83 million people in Germany have no Internet connectivity (D21 Index 2020), both the quality and the quantity of data transmission is, in many cases, inadequately accessible, either because the infrastructure for high-speed transmission is absent (Dalg 2020) or because data rates continue to make access unaffordable for some learners (Drabe 2020; Kutter 2020). In such instances, the first-level digital divide is the basis for the mismatch between teachers and their pupils that may lead to widening spirals of difference. Robinson (2009) describes how learners with unfettered, adequate digital access can explore, take risks, and leisurely pursue information online, whereas other learners are forced to become highly concentrated, ‘goal-oriented agents’ with narrow foci and purposeful use of the tools. Robinson (2009) concludes that this latter situation gives rise to a ‘taste for the necessary’ (491) and a lack of autonomy, highly prized and expected in German educational environments (Bender-Szymanski 2000; Levesque et al. 2004; Reusser 2006) and identified as one of the factors leading to learner participation during the school closings in 2020 (Fischer et al. 2020; Huber and Helm 2020). It is important to note that, while Robinson’s (2009) study focused on learners with limited financial means that inhibited middle-class learner behaviors online, in Germany, many families’ access is constrained, not by (just) wealth, but by inadequate infrastructure. While these limitations might be negotiable in typical scenarios by using publicly available Internet, during the Covid-19 period access to libraries, schools, and public transportation was severely curtailed. It remains to be seen how the behaviors of learners in highly educated and financially secure families will be informed by these conditions.

### Digital Cultural Capital

Regardless of economic capital, both teachers and families possess digital skills that offer them, in their respective fields, substantial cultural capital. However, there is frequently a mismatch between these skills and those needed to carry out digitally mediated schooling effectively (Sliwka and Klopsch 2020). While the habitus of teachers has led to a focus on minimalistic digital skills for informational and communication purposes, the habitus of many learners is centered on leisure usages of these technologies. This has led to substantial tensions between the teachers and learners, and among parents asked to support their children in their academic digital forays. On the one hand, teachers have expressed frustration on social media regarding students who repeatedly forget log-in information or who do not know how to attach documents to e-mails (Brimer 2020; Wombat 2020a, 2020b). On the other hand, students—and their parents—have voiced resentment over the multitude of tools they need, worksheets in poor visual quality, and copious printing and scanning requirements (Diekmann 2020; Huber et al. 2020; Peters 2020; Wacker et al. 2020) with materials that often represent little more than digital versions of traditional worksheets, more or less adapted for the current situation (Köller et al. 2020). These experiences echo the findings of more representative studies from before the school closings that attest to limited educationally oriented digital competency among teachers and learners (Eickelmann and Drossel 2020). That cultural capital and economic capital go hand-in-hand is obvious: teachers and school leaders with similar backgrounds, with a habitus that does not value digital teaching and learning, do not necessarily advocate for digital tools in their schools. Likewise, many learners have little idea how their leisure pastimes could be transformed into institutional, educational capital.

Given the aforementioned habitus, there are numerous teachers with digital skills that allow for only limited digitally mediated pedagogy and communication. While 80% of teachers report using email to stay in personal contact with their students, the nature of this contact is unclear (Arp et al. 2020; Huber et al. 2020). Beyond email, there are widespread disparities in how teachers mediated schooling during the school closures, or how receptive they will be to doing so in the future. A 2019 study suggests that more than half of teachers in Germany are willing to use more digital media in their instruction. However, they simultaneously report the need for more professional development and ambivalent attitudes towards the use of digital tools (Rohleder 2019). These issues of limited knowledge and ambivalent attitudes may be related: 70% of the teachers surveyed suggested that the quality of available materials for digital learning was poor. This indicates that knowledge of tools with which teachers can develop their own materials, competence-oriented usages of authentic web offerings, and practices such as adapting open educational resources (OER) to meet situated needs, is not widespread. Given the ways in which some of these practices require reflection of more broadly fundamental philosophies, such as constructivist learning, critical pedagogy, and collaborative professional practice, the need for professional development opportunities based on empirically based principles of professionalization regarding a range of pedagogical issues is tantamount.

In addition to issues of access and competence, structural issues have played a predominant role in the emerging discourse, especially as regards devolved control of schools and teachers’ pedagogical autonomy, emerging from, among other things, historical concerns regarding authoritarianism (Kerres 2020). Likewise, another area of increasing concern regards privacy and security issues, with inconsistent interpretations of European data privacy laws generating confusion and a reluctance to use many tools for fear of legal repercussions. An issue that has thwarted digitalization in Germany’s schools for years has only become more intractable in light of recent events (Fokken 2020b; irights 2020; Kerres 2020). This discourse is not merely a legal or ethical one; attitudes towards data privacy informed by its ignominious past play a significant role in how this issue in particular is enacted in Germany. These are further complicated by attitudes towards, and consequences of, legal violations.

## A Look Ahead: A Pessimistic Perspective

Despite the widely acknowledged problems that have become more visible since mid-March of 2020, it is unclear what the implications will be moving forward. While there are some indications that the experiences documented during the period during which schools were closed may lead to relatively radical leaps forward in terms of digital infrastructure and training, the impact on teachers’ and learners’ attitudes is less clear. There are some indications that, for some teachers and learners, the crisis has provided them with an opportunity to broaden not just their practices, but also their ‘tastes.’ For others, the challenges of the preceding months may deepen their skepticism.

The potential of teachers emerging from this crisis with greater digital competence and even greater distaste of digitalization is significant. Forced to transition to emergency remote teaching with inadequate preparation instigated a ‘steep learning curve’ (Kerres 2020) for many that, due to its chaotic and urgent nature, left participants with little opportunity to develop concepts that reflect underlying pedagogical principles. Frustration with the implementation of approaches and tools that do not achieve the desired results, such as engaging students, building knowledge, or strengthening subject-related competencies, may lead to a rejection of digitally mediated teaching in the future. This is especially a potential consequence in light of the cognitive, financial, and time resources teachers invested in identifying appropriate applications, learning the technical skills needed to use new media, or developing materials that integrate or rely on these tools.

At the other end of the spectrum, there may be teachers who have discovered a ‘taste’ for digital tools and enthusiastically plunge ahead, without taking the time to develop and implement pedagogically meaningful concepts (Warnecke 2020). While the mantra of ‘just do it’ has both supporters and opponents in regard to digitally mediated teaching and learning, the risks of pedagogically problematic practices associated with this approach are high (Krommer 2018a). Bär (2019) argues that using digital tools without first considering the pedagogic goals and the affordances of the media can only be a type of didactic experiment, following which a critical reflection is necessary to ensure the usage’s value, identify the reasons for success or failure, and embed the methodological approach in a meaningful instructional aim. Whether this reflection can and will take place in the current situation and given the current structures is questionable (Muuß-Merholz 2020), especially in light of the fact that schools have now been advised to prepare for a return to a traditional model of schooling following the summer break (Fickermann and Edelstein 2020). This decision, which ignores the expert commission on education and schooling’s recommendations from April 2020 to prepare for three eventualities ranging from completely distanced learning, a mix of presence and distance learning, and online learning (Maaz and Jungkamp 2020), leaves teachers and teacher leaders with little time to conceptualize new approaches and even less incentive to do so.

The danger that the shift to digital tools will remain simply a substitution of tools and not a reconceptualization of the nature of schooling to reflect the ways in which these tools can facilitate individualized, interactive, spatially and temporally unbounded learning also looms (Chou and Block 2019). This focus on the medium, rather than the ways in which the medium changes how people learn and interact, ignores the culture of schooling altogether. Such an approach to digital teaching and learning would be commensurate with, as Muuß-Merholz (2020) puts it, using the tools of the twenty-first century to optimize the educational ‘grammar of schooling’ of the nineteenth and twentieth centuries. Hoffmann (2020) goes so far as to posit that this is exactly the goal of many educational policymakers, arguing that the prospect of revolutionary change is in part the reason for strident calls for an expeditious return to normalcy.

Finally, given the challenges German teachers have had addressing heterogeneity—or even individualization—even before the Covid-19 outbreak (Combe 2005; Reh 2005), there is a danger that their attempts at digitalization will falter by not reflecting adequately the individual needs of various learners. One of the groups of students most significantly affected by the school closing was that of learners with special educational needs (Fokken 2020a). Excluded in many cases from online learning opportunities because of necessary accommodations, these learners are among those for whom the return to the physical classroom is often structurally most challenging (Hanack and Kabel 2020; Wrase 2020). In some cases, students have complex medical needs that make them susceptible to infection. In other cases, their cognitive or physical handicaps are perceived as incompatible with schools’ hygiene plans (Hanack and Kabel 2020; Knödler 2020). Moreover, teachers who rely on the support of special educational personnel for individual students have not had the opportunities to collaborate with these professionals in ensuring that digitally mediated activities and tools are accessible (Die Neue Norm 2020). Although digitalization can contribute to inclusion, by, for example, enabling students with disabilities to participate with assistive technologies or via distance communication, ensuring the accessibility of learning platforms and learning management systems has been neglected. The difficulties connected with meeting these learners’ needs, as well as the diverse needs of learners with differing language skills, prior knowledge, learning behaviors, and digital access, may prove too difficult to manage.

## A Look Ahead: An Optimistic Perspective

Despite the challenges enumerated in the preceding section, it is not necessarily the case that these forebodings will come to pass. The current data cannot yet provide insight into which trends will emerge as more representative than others. In many cases, attitudes are complex and cannot easily be summarized with quickly generated quantitative data, but rather necessitate more time-consuming qualitative processes in order to be penetrated (but see: Reischl and Schmölz 2020). However, there are some indications that those same digital tools eyed partially with skepticism by many teachers also provide others with opportunities. One area where this is particularly noticeable is in the highly significant domain of collaboration and co-construction of teaching knowledge (cf. Sliwka and Klopsch 2020). Many such conversations take place on Twitter, and are documented with the hashtags #*twitterlehrerzimmer* or #*twlz*, which serve as computer-mediated personal learning networks (Bündgens-Kosten and Kemmerer in press; Nölte 2017). In this way, the experiences of the spring semester 2020 could serve, for some, as ‘the most effective professional development measure of the century’ (Schratz 2020).

Both here and in other networks, those teachers who have embraced digitalization and who embody a different set of beliefs have been able to use the Covid-19 closings as an opportunity to demonstrate effective digitally mediated pedagogies and support colleagues and learners in their digital forays. Their activities range from demonstrations of best practices and descriptions of digitally mediated teaching and learning in their own classrooms to support in using individual tools and recommending collections of programs and applications. Others emphasize didactic principles of digitally mediated teaching and learning or provide support for infrastructure development. Many of these individuals explicitly connect theory and practice (Bankhofer et al. 2020; B. Blume 2020a; Krommer et al. 2020; Vedder 2020a).

Other opportunities may arise through the structural changes that have been introduced as a result of the school closings that have, at first glance, little to do with digitalization per se. While the German federal states unanimously agreed to carry out the school-leaving exams in challenging conditions (for an analysis of how federalism impacts digitalization in German schools, see Kerres 2020), consensus regarding other long-held practices has declined. Some of the federal states have eliminated the practice of retaining students in a grade due to poor performance, and others have suspended *Abschulung*, in which students in more academic school forms are relegated to less challenging vocational or trade schools. Others have granted schools and teachers additional leeway in assessing content delivered during the period of school closings, and have allowed for alternative assessment procedures, types, and number of exams (Olbrisch 2020; Süddeutsche Zeitung 2020; Unterberg 2020a). These accommodations have given rise to changes in both pedagogical and digital practices (Busch 2020; Förtsch 2020b; Vedder 2020b); they have led to reflection regarding entrenched expectations and the interrogation of ingrained dogmas that, at their core, question assumptions about the nature of schooling and education in the twenty-first century and thus implicitly or explicitly address beliefs about digitally mediated teaching and learning (Burow 2020; Maaz and Jungkamp 2020; Sliwka and Klopsch 2020).

These changes may have a snowball effect, leading to other benefits. Already, some analysts and parents have recognized that, for some learners, the ‘new way’ of doing things has proven advantageous (Fleming 2020; Fugmann 2020; HaVaRi 2020). They name the opportunities for autonomous and creative learning at their own speed as some of the benefits of distanced learning (Huber and Helm 2020). Moreover, digital tools may be able to better engage learners and families who were previously marginalized by monolinguistic and traditional school norms that have been criticized for being unwelcoming towards those with less academic backgrounds (Fugmann 2020; Gomolla 2009). Some teachers have already acknowledged that their ability to diagnose students’ learning needs, establish personal relationships, and individualize instruction has been enhanced by the implementation of digital tools (Förtsch 2020a). Structural alternatives that shift from an equalities-based approach to an equity-based approach have also been advocated (Bremm and Racherbäumer 2020; Goldan et al. 2020; Wrase 2020).

Further impetus can be found as regards digitalization directly. While the digital investment pact of 2019 afforded schools 5 billion Euros to promote digital tools and infrastructure has been criticized for its bureaucratic nature and inability to affect meaningful change (Scheller 2019), monies in the Covid-19 emergency fiscal package to finance system administrators in schools heralds acknowledgement of a long-abiding demand (bpv 2020; D64 2020). Recommendations of educational researchers who advocate universal access for learners, better infrastructure, and thoroughgoing professional development opportunities have likewise gained increased attention (Maaz and Jungkamp 2020). Whether this sudden surge in interest will lead to increased funding and sustained investment remains to be seen, but it does indicate an awareness that the postdigital nature of contemporary societies (Jandrić et al. 2018) can no longer be ignored.

## Implications for Teacher Education

In order to avoid a crisis of legitimacy, the habitus of schools and that of teachers need to reflect the lessons learned in the wake of the Covid-19 health crisis. While Germany’s other political and social systems fared, by many measures, comparatively well in the initial pandemic wave, the challenges faced by its educational system were crystallized. With the current consensus regarding the need for both strengthened digital infrastructure and digitally mediated pedagogy, the question emerges as to how teachers can be supported in the development of effective pedagogical practice in a transformed society. Although many educators in Germany were forced by circumstance to adapt and adopt to a situation for which most of society was ill-prepared, there is no universal consensus that the weeks of digital instruction afforded advantages over traditional methods of teaching and learning. Nor is there any guarantee that the emergency solutions taken during the crisis can be transformed into lasting professional growth. Thus, the question arises as to what teacher initial education and professional development need to transpire in order to ensure that the current momentum is not lost.

While educational researchers have been expounding on specific measures for years, their recommendations gain new urgency in light of the recent past. In addition to digital hardware and software, financed or subsidized by public authorities, teachers need opportunities for professional development. This includes an examination of existing school structures, which may require reform to facilitate embedded and universal professional development that is a part of teachers’ professional time (Darling-Hammond et al. 2017). Moreover, this further education needs to reflect empirical findings regarding professional development: it needs to address both beliefs and behaviors, be embedded in subject-matter didactics and teachers’ situated environments, and connect theory with practice and teachers with one another as learners (Biedermann et al. 2012; Daschner and Hanisch 2019; Lipowsky and Rzejak 2019).

Methodologically, professional development must model the kinds of learning teachers should utilize with their own students (Wahl 2013). This entails, firstly, taking advantage of digital approaches that can inform, individualize, personalize, support interaction, facilitate relationships, and provide feedback (Redecker 2020). Simultaneously, teachers should be guided, by connecting new input with opportunities for practice, feedback, and reflection (Lipowsky and Rzejak 2019), into considering the ways in which digital media can, given these affordances, re-align existing hierarchies (Heidkamp and Kergel 2019). Initial debates regarding preferences for synchronous or asynchronous teaching or whether learners should be required to turn on their webcams or not can provoke deeper conversations about access, equity, and safety. A more formalized approach to facilitating reflection is to incorporate video-based observations or analyses among small groups of peers. Repeated empirical studies have demonstrated the role of scaffolded reflection with videotaped lessons of oneself and of others (Brouwer 2014). While videotaping instruction during a future phase of school closures will necessitate adaptation to changed spaces and forms of teaching and learning, this very novelty may make the idea both more palatable and easier to pragmatically manage. These could be utilized in part to, while ensuring psychological and professional safety, create moments of cognitive dissonance that may lead to conceptual change (Anderson et al. 2018; Combe 2005).

Although evidence suggests that it is difficult to change underlying beliefs, there is also evidence that this can be accomplished (Biedermann et al. 2012; Blume et al. 2019). Such transformations need to be initiated and sustained, not just as regards digitally mediated teaching and learning, but regarding the nature of effective pedagogy more generally. For example, teachers have shared the ways in which the shift to online teaching has changed their planning, interaction patterns, and feedback methods. Superficially related to the transition online, all of these themes relate fundamentally to their underlying assumptions about the choreography of teaching and learning more broadly, and are being called into question in this time of upheaval. While attitudes deriving from habitus, both personal and professional, are acquired implicitly and over decades, they are not immutable (Mouzelis 2008). Indeed, the multidimensionality of habitus itself lends itself to critical reflection (Bolten 2018). It is especially important to understand the ways in which these beliefs both about technology and about schooling, informed by habitus, and the technology itself, interact. For example, while 73% of secondary students surveyed last year indicated that digital media could be used to support the individual needs of diverse learners, for example, only half of the teachers recognized this potential role of digital tools (Hubig and Berg 2020; Rohleder 2019), and only 15% of teachers in 2018 actually used digitalization in these ways (Eickelmann and Gerick 2020). Although more data would be necessary to fully examine this discrepancy, it suggests that the teachers and students perceive different affordances as a result of digitalization. It also raises questions about other issues surrounding cultures of teaching and learning, such as differentiation in light of increasing heterogeneity. Thus, the mismatch goes beyond attitudes towards, and knowledge of, digitalization, but also touches on questions regarding the appropriate and effective nature of instruction more widely.

## Conclusions

Habitus alone does not account for the lack of digitalization found in German schools in 2020. However, by examining the situation through the lens of habitus, it is possible to gain a deeper understanding of the circumstances that existed as schools rushed to close in March 2020. This perspective also helps to contextualize the experiences made by both teachers and students in the subsequent weeks. While further empirical and theoretical analysis is necessary, it is possible to understand the tensions between teachers and learners (and their proxies) in light of mismatches in digital cultural capital, emerging in part as a result of the personal and professional habitus of teachers.

Despite widely differing beliefs and attitudes, students and teachers agree that Germany’s schools are lagging behind when it comes to digital learning concepts, methods, and tools (Rohleder 2019; Huber 2020; Eickelmann et al. 2019). Given the heightened interest in the situation of schooling during a time when digital tools provided the only access to education, a broad consensus has emerged in this regard that infrastructure and skills have both been neglected. While considering the reasons for this state of affairs is an important first step, anticipating further developments, both in terms of the attitudes of the institution’s participants and as regards future opportunities to learn both during and beyond the pandemic, is necessary if the experiences articulated in the heat of the moment are to lead to new outcomes. These analyses need to cast a wide net, extending far beyond the narrow confines of digital tools to examine the habitus of schools and the role of the participants in these schools more broadly. In an illustrative example of one of the first qualitative analyses of teachers in Germany during Covid-19 conditions, Reischl and Schmölz (2020) describe how a teacher sees himself as the antidote to parental exasperation: Parents are thoroughly overwhelmed, despite digital educational materials, in the teacher’s absence. He [sic] alone in his exalted stature as pedagogical expert can rectify the situation. In this case, addressing this teacher’s digital efficacy would fall short of bringing about a critical understanding of the changing nature of schooling post-Covid-19. Rather, his digital attitudes need to be contextualized within the broader structure of his teaching personae, institutional role, and conceptions of the nature of subject-matter knowledge and subject-specific pedagogy.

Understanding the habitus of teachers and the ways in which it mediates pedagogy in general, and digitally enhanced teaching and learning more narrowly, provides educational leaders and policymakers opportunities to affect the environment in which the habitus is enacted. While a pre-existing familial habitus cannot be altered, but at most critically reflected upon, the opportunity to use such reflective approaches to change patterns of schooling in the wake of Covid-19 is greater than ever before. The next generation of teachers—even though they are likely to be, at least for the time being, from similar milieu as current educators—will come of age in a radically different personal, academic, and professional environment. Whether this new state of affairs will lead to habitus more welcoming of a wider range of digital behaviors remains to be seen, but the potential for significant ruptures with entrenched cultures is high, especially if the pandemic leads to further school closures. Teacher educators at the university level and in subsequent phases of teacher preparation will need to consider the ways in which the attitudes of these students towards digitally mediated schooling have been forged in a time of broader paradigmatic crisis and rapidly changing norms against the background of stable and conservative habitus. In Germany, ideas regarding the management of radical transitions in educational contexts could draw specifically on those lessons learned in the wake of the collapse of East Germany, which saw all members of the school community grappling with new ‘ways of being’ (Dall’Alba 2009: 34) and which necessitated a close examination of primary and secondary habitus of teachers and learners (Helsper et al. 2001).

However, as teachers report feeling increasingly burdened by teaching in classes with stringent hygiene rules that require alternative pedagogies, increases in their contact hours through the creation of smaller groups, and the obligation to offer online resources for learners who are unable to attend school (Allen et al. 2020; GEW 2020b; Teacherlise 2020), it is imperative that existing and new impositions on them are considered. Teachers who are overwhelmed are less likely to be able to meaningfully reflect on the reasons for this state of affairs and address potential gaps in their competences that increase these burdens, especially as they require critical distance from their own norms. Any attempts to address the nature of teaching and schooling in a postdigital society will require examination of long-held and deeply situated personal and systemic beliefs, a process that requires a receptivity to grappling with challenges to these pre-existing assumptions (Helsper 2018). Anticipating that educators can carry out this kind of soul-searching while functioning at their physical and psychological limits would be unrealistic (Margolis and Nagel 2006). Calls to ‘return to normalcy’ despite an unchanged public health situation coupled with complex hygiene guidelines minimize their existential and professional concerns, and eliminate any opportunities to facilitate transformations.

Public and professional recognition of the challenges faced by German educational institutions and educators in the wake of the Covid-19 crisis has led to an influx of monetary resources and political attention, the latter of which has not always been sympathetic to teachers’ perceived struggles. This discourse in itself potentially magnifies the problems faced by individuals and systems ill-equipped to face radical and rapid change. One of the more destructive aspects of these analyses is their tendency to ascribe negatively connotated motivations to individuals without recognizing the sociocultural influences that inform their habitus (Füller 2020; Mink 2020; Unterberg 2020b). These decontextualized demands for transformation are unlikely to be successful without equal attention to the beliefs that led to this situation. Given the foundational nature of teachers’ habitus in constructing these beliefs and as the mode through which these can be altered (Hardy and Melville 2013), ignoring the habitus may be one of the riskier responses to addressing post-pandemic pedagogy.

## Data Availability

The cited references can be made available upon request. No uncited or unpublished data or material other than the author’s has been included.

## Footnotes

1 Whereas Eickelmann and Drossel (2020) found that one-quarter of teachers in Germany had access to a learning management system, Huber et al. (2020) found 43% of German teachers attested to the availability of a learning platform. The discrepancies highlight the emerging nature of the data and may be due to different wording, as Eickelmann and Drossel focused specifically on learning management systems, whereas Huber et al. subsumed these in the more general category of learning platforms.
